# Facilitating Web-Based Collaboration in Evidence Synthesis (TaskExchange): Development and Analysis

**DOI:** 10.2196/resprot.9285

**Published:** 2018-12-13

**Authors:** Tari Turner, Emily Steele, Chris Mavergames, Julian Elliott, Project Transform Team

**Affiliations:** 1 School of Public Health and Preventive Medicine Faculty of Medicine, Nursing and Health Sciences Monash University Melbourne Australia; 2 Cochrane Central Executive Freiburg Germany; 3 Department of Infectious Diseases Monash University and Alfred Hospital Melbourne Australia

**Keywords:** review, systematic, intersectoral collaboration, software, internet

## Abstract

**Background:**

The conduct and publication of scientific research are increasingly open and collaborative. There is growing interest in Web-based platforms that can effectively enable global, multidisciplinary scientific teams and foster networks of scientists in areas of shared research interest. Designed to facilitate Web-based collaboration in research evidence synthesis, TaskExchange highlights the potential of these kinds of platforms.

**Objective:**

This paper describes the development, growth, and future of TaskExchange, a Web-based platform facilitating collaboration in research evidence synthesis.

**Methods:**

The original purpose of TaskExchange was to create a platform that connected people who needed help with their Cochrane systematic reviews (rigorous syntheses of health research) with people who had the time and expertise to help. The scope of TaskExchange has now been expanded to include other evidence synthesis tasks, including guideline development. The development of TaskExchange was initially undertaken in 5 agile development phases with substantial user engagement. In each phase, software was iteratively deployed as it was developed and tested, enabling close cycles of development and refinement.

**Results:**

TaskExchange enables users to browse and search tasks and members by keyword or nested filters, post and respond to tasks, sign up to notification emails, and acknowledge the work of TaskExchange members. The pilot platform has been open access since August 2016, has over 2300 members, and has hosted more than 630 tasks, covering a wide range of research synthesis-related tasks. Response rates are consistently over 75%, and user feedback has been positive.

**Conclusions:**

TaskExchange demonstrates the potential for new technologies to support Web-based collaboration in health research. Development of a relatively simple platform for peer-to-peer exchange has provided opportunities for systematic reviewers to get their reviews completed more quickly and provides an effective pathway for people to join the global health evidence community.

## Introduction

### Background

Peer-to-peer Web-based marketplaces are designed to simply, quickly, and reliably connect people who need a service or product with people who can provide it. Services like Etsy, Airbnb, and Airtasker are already familiar to many people, and others are constantly being developed to cater to different sectors or groups in society [1].

In parallel with these developments, the conduct and publication of scientific research is becoming increasingly open, international, and collaborative [2-4]. For example, in the decade between 1990 and 2000, the proportion of scientific papers that were published by international collaborations doubled to almost 16% [4]. In the same period and subsequently, there have also been substantial developments in Web-based collaboration technologies. As a result, there is growing interest in Web-based platforms that can enable global, multidisciplinary networks of scientists in areas of shared research interest [5]. Platforms like ResearchGate, Academia, and LinkedIn provide professional networking opportunities across and beyond science. Other platforms like Benchling (molecular biology), Kaggle (data science), and nanoHub (nanotechnology) provide access to a range of tools, networks, and resources relevant to specific scientific fields.

TaskExchange [6] is an example of a Web-based collaborative platform in biomedical science. The aim of TaskExchange is to bring together people who need help with their systematic reviews and other forms of research synthesis (task posters) with people who have the time and skills to help (task responders), thereby facilitating efficient production of high-quality, relevant, up-to-date evidence syntheses to inform health policy and practice. From the outset, task posters were envisaged as being leaders and project managers of health evidence projects, while task responders would be altruistic individuals, such as retirees, or people seeking health evidence skills, such as medical students. Task posters are able to offer task responders authorship or acknowledgment in project outputs as well as a monetary reward, as deemed appropriate. This paper describes the rationale for TaskExchange and the processes involved in developing and running the platform. Data are presented to describe the use and effectiveness of the platform to date.

### Systematic Reviews and Cochrane

Systematic reviews collate and synthesize evidence from research to support the best possible health care decisions and are a key step in the translation of the results of research into improved health care practice [7,8]. Producing high-quality, relevant systematic reviews and keeping them up to date requires substantial resources, skill, and time [9,10].

Cochrane has been producing systematic reviews for more than 20 years and is a leading producer of systematic reviews of health care research. The Cochrane Database of Systematic Reviews includes more than 7000 systematic reviews, and another 2500 reviews are in development [11]. Cochrane reviews are prepared by author teams who work with 1 of 52 Cochrane review groups responsible for a specific area of health care or policy. Author teams are often international in composition and can include clinicians, consumers, and researchers with a range of experience and skills working together.

Cochrane reviews and other research syntheses increasingly require input from contributors with specialist skills outside the immediate author team. Examples include translation (as Cochrane reviews include research published in multiple languages), specialist methodological input from expert statisticians, and input from health care consumers to ensure the relevance and usefulness of the reviews. Cochrane is committed to enabling diverse and inclusive input into the formulation, production, and dissemination of Cochrane reviews, seeking to ensure they meet global needs for high-quality evidence synthesis.

The increasing recognition of the importance of diverse contributions to the work of Cochrane, combined with growing interest from people to get involved in systematic reviews, led to the development of TaskExchange, a Web-based collaboration platform, to support the conduct and uptake of systematic reviews of health research evidence.

## Methods

### Platform Goals

The original aim of the development of TaskExchange was to create a platform that connected people who needed help with their Cochrane reviews with people who had the time and expertise to help. The initial concept included three major components:

1. Creation of a directory of experts in elements of the systematic review process.
2. The ability for members to post tasks that described a need they had for help.
3. The ability for members to respond to tasks that were in areas of their interest and skill.

### Software Development

**Figure 1:**
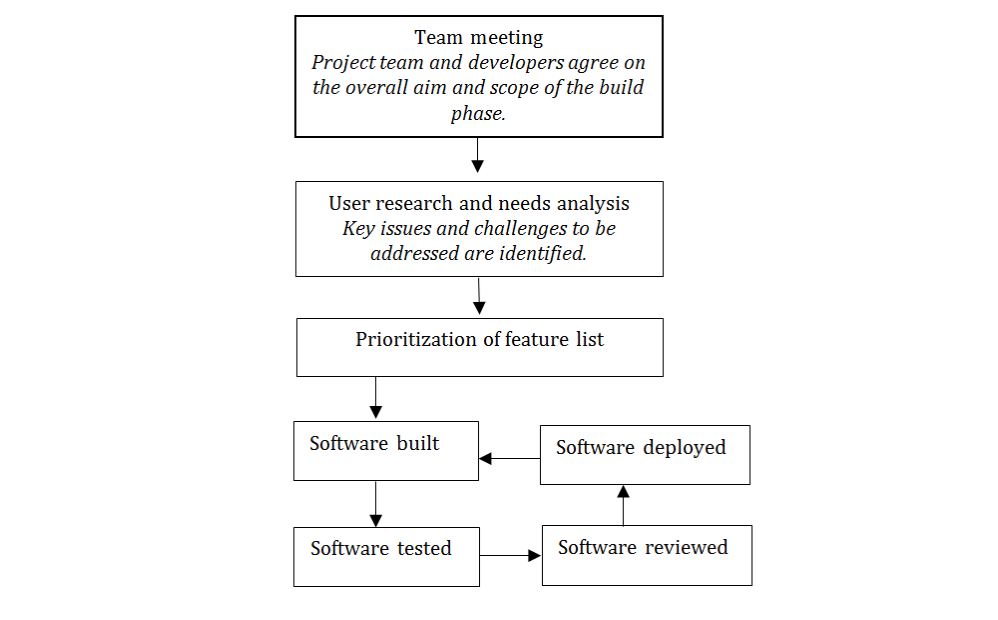
Development process.

The development of TaskExchange consisted of 5 development phases using an agile approach, which included iterative development with frequent releases of new software and close collaboration between the development team and the project team [12]. Each development phase involved a sequential process, as shown in Figure 1. Software was iteratively deployed (made available live to users) as it was developed and tested, enabling close cycles of development and refinement. The platform was built by a software development company (Cogent) using Ruby (a programming language) and Ruby on Rails (a server-side Web application framework written in Ruby) and JavaScript (a programming language) on a server hosted by Heroku (a container-based cloud Platform as a Service). We built simple mechanisms for tracking user numbers, numbers of tasks posted, and response rates to posted tasks using data clips to display the results of structured query language queries on a Heroku database.

### Development of TaskExchange

As described above, the development of TaskExchange was undertaken in 5 phases over 2 years. Table 1 describes each phase of development.

**Table 1:** Description of TaskExchange development phases.

Phase	Start date	Release date	Purpose	Summary of functions added
1	July 2015	October 2015	Test whether a simple, well-designed Web-based platform could help enable collaboration within the Cochrane community by connecting people who needed help with their review with others who had the time and skills to help.	Minimal functionality, allowed users to create a profile and post and respond to simple tasks.
2	December 2015	February 2016	Refine initial designs and develop a more fully featured prototype.	Improved task classification and addressed issues that had arisen in user testing.
3	April 2016	August 2016	Develop a more user-friendly, open-access platform.	Improved the matching of translation tasks by enabling users to specify required language skills. Improvements made to the way user profiles were created and displayed. TaskExchange opened to the public (it had previously been restricted to people with Cochrane accounts).
4	December 2016	June 2017	Add key features and broaden the scope of TaskExchange.	This phase focused on building features that enable users to endorse people for their skills and acknowledge their work. It also broadened the scope of the tasks within TaskExchange to include those relevant to the development of clinical practice guidelines.
5	January 2018	April 2018	Streamline the use of the platform for users.	This phase focused on creating a new dashboard within the platform for all users, called “My Tasks.” Users can manage all tasks they have posted and respond to within the MyTask tab.

### Support and Community Engagement

After the release of the open-access platform (Phase 3 in Table 1), a part-time community engagement and partnerships manager (CEPM) was employed to oversee, implement, evaluate, and refine the Web-based community engagement strategy for the TaskExchange platform. The role aimed to increase user numbers, tasks posted, and tasks matched. We initially focused on building engagement with key brokers within the Cochrane community, including Managing Editors, and also areas where existing informal networks for finding help with systematic reviews were weakest (eg, translation and consumer networks). For each stakeholder group within Cochrane, the CEPM worked with key champions to design a purposive engagement strategy including tactics such as webinars, articles in newsletters, and blogs and articles for group webpages.

The CEPM also aimed to engage people beyond those already working with Cochrane. Other organizations that focused on producing systematic reviews were engaged using similar tactics as those used with the Cochrane stakeholders. Additionally, social media, in particular, Twitter, was used to engage a broader audience, with strategic use of hashtags and identification of relevant Twitter champions who could help spread the message of TaskExchange. For TaskExchange members, the CEPM provided a point of contact and user support, capturing issues and opportunities for further platform development.

## Results

### Current Functionality of TaskExchange

The current version of TaskExchange is available on the Web [6]. The site is still in active testing and development. Multimedia Appendix 1 shows key webpages from the TaskExchange platform. Table 2 shows key features of the platform and dates when features were released.

TaskExchange also has administrative functions, including the ability for administrators to edit tasks and view usage data.

### Growth of TaskExchange

**Figure 2:**
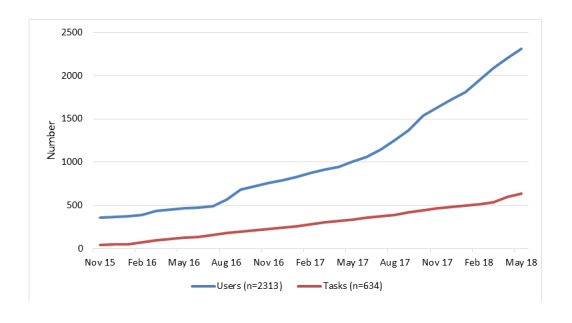
Number of TaskExchange members and tasks, November 2015-May 2018.

Between November 2015 and May 2018, TaskExchange hosted 634 tasks and gained 2313 members. Figure 2 shows the monthly growth of tasks and members. Currently, around 40 tasks and 30 users are added per month (data are averages from the 3-month period of March-May 2018). Approximately 50% of TaskExchange members did not have an existing connection to Cochrane before joining TaskExchange.

### TaskExchange Users

Table 3 describes TaskExchange users by global distribution, user type (poster vs responder), and skills. As shown in the table, 24.25% (561/2313) of the users are from Europe and a little over 10% (282/2313, 12.19%) are located in Asia. The geographical location of 41.98% (971/2313) of users is unknown, with these users opting not to share that information when signing up to TaskExchange. Only 34.37% (795/2313) of users have actively engaged on TaskExchange: 9.77% (226/2313) as posters, 22.96% (531/2313) as responders, and 1.64% (38/2313) as both posters and responders.

TaskExchange members have a broad array of skills. Members can nominate one or more skills from a predefined list that was compiled following consultation with the intended user community in early platform development. As seen in Table 3, the most common member skills are data extraction (426/2313, 18.42%) and literature screening (366/2313, 15.82%).

### Types of Tasks Posted

Table 4 shows the number and types of tasks that have been posted on TaskExchange up to May 2018. Translation accounts for 49.8% (316/634) of all tasks, followed by data extraction (145/634, 22.9%) and application of inclusion or exclusion criteria (122/634, 19.2%).

**Table 2:** TaskExchange features and release dates.

Feature	Release date
Sign in (by creating a TaskExchange account)	February 2016
Sign in (by creating a Cochrane account)	January 2018
Create a personal profile describing member skills and experience	February 2016
Browse and search tasks using keywords or nested filters	February 2016, subsequently improved
Browse members using keywords or nested filters	February 2016, subsequently improved
Respond to tasks that interest members by sending a message from within TaskExchange	February 2016
Post tasks describing the nature and timelines of the task, the skills required, and reward offered	February 2016
Choose a responder appropriate for the task	February 2016
Unpublish tasks for which a responder has been found	February 2016
Report whether a task responder was found via TaskExchange	February 2016
Sign up to weekly task notification emails	February 2016
Endorse and acknowledge the work of TaskExchange members	June 2017
Manage all tasks posted and responded to from a central place (My Tasks)	April 2018
Report when a task has been completed	April 2018

**Table 3:** Description of TaskExchange users (N=2313).

Descriptive variable			Value, n (%)
**Global distribution of users**			
	Africa		62 (2.68)
	Antarctica		0 (0)
	Asia		282 (12.19)
	Australiana		119 (5.14)
	Europe		561 (24.25)
	North America		196 (8.47)
	South America		122 (5.27)
	Unknown		971 (41.98)
**User type**			
	Poster		226 (9.77)
	Responder		531 (22.96)
	Both		38 (1.64)
	Neither		1518 (65.63)
**User skills^a^**			
	Translation		296 (12.80)
	Consumer input		72 (3.11)
	Data extraction		426 (18.42)
	Clinical input		180 (7.78)
	Protocol development		258 (11.15)
	Qualitative analysis		159 (6.87)
	Question formulation		181 (7.83)
	Report writing		309 (13.36)
	**Review**		
		Clinical content	189 (8.17)
		Consumer	56 (2.42)
		Knowledge translation	114 (4.93)
		Copyedit	174 (7.52)
		Methods	204 (8.82)
		Prioritization	34 (1.47)
	Risk of bias assessment		269 (11.63)
	Screening		366 (15.82)
	Searching		315 (13.62)
	Statistical analysis		217 (9.38)
	Summary of findings table		204 (8.82)
	Other		10 (0.43)

^a^Users can nominate more than one skill.

**Table 4:** Type of task posted on TaskExchange and response rate per task type.

Type of task^a^		Total tasks^b^, n (%)	Response rate per task type (%)
Translation		316 (49.8)	71
Consumer input		101 (15.9)	49
Data extraction		145 (22.9)	69
Clinical input		36 (5.7)	50
Inclusion or exclusion criteria		122 (19.2)	67
Protocol development		43 (6.8)	77
Qualitative analysis		16 (2.5)	81
Question formulation		26 (4.1)	81
Report writing		30 (4.7)	90
**Review**			
	Clinical content	36 (5.7)	69
	Consumer	66 (10.4)	38
	Knowledge translation	18 (2.8)	83
	Copyedit	17 (2.7)	53
	Methods	32 (5.0)	91
	Prioritization	1 (0.2)	100
Risk of bias assessment		57 (9.0)	56
Screening		47 (7.4)	70
Searching		41 (6.5)	80
Statistical analysis		30 (4.7)	70
Summary of findings table		25 (3.9)	76
Other		1 (0.2)	100

^a^Tasks can be categorized according to one or more of these categories.

^b^Total number of tasks, N=634.

**Table 5:** Rewards offered to responders and response rate per reward type.

Type of reward	Total tasks^a^, n (%)	Response rate per reward type (%)
Payment	23 (3.6)	65
Authorship	86 (13.6)	72
Acknowledgment	481 (75.9)	63
No reward	79 (12.5)	68

^a^Total number of tasks, N=634.

Table 5 shows the distribution of rewards offered to task responders. Task posters can allocate one or more rewards per task from a set list that includes payment, authorship, and acknowledgment. They may also elect not to offer a reward. Table 5 shows that 75.9% (481/634) of tasks offer acknowledgment to the responder and only 3.6% (23/634) of tasks offer payment to the responder.

### Task Responses

The response rate for tasks posted between the middle of August 2016 (when the site became openly accessible) and May 2018 was 78.1% (495/634), that is, 78.1% of tasks posted within that period received at least one response.

Table 4 shows the response rates per task type. Rates vary considerably across type, ranging from 38% (25/66) for consumer review to 100% for review prioritization, although only 1 task of the latter type has been posted. Table 5 shows that response rates vary little with the type of reward offered; 63.0% (303/481) for acknowledgment, 72% (62/86) for authorship, and 68% (54/79) when no reward is offered.

### Task Matching and Completion

As with other peer-to-peer marketplaces, collecting accurate data on matching and completion is challenging and relies to some extent on task posters marking tasks as matched or completed. Also, data are difficult to interpret because of variability in the nature and duration of tasks. For example, some tasks require more than one appropriate responder, and tasks vary in time to complete from as little as 5 minutes to tasks with no natural endpoint (eg, authorship on a Cochrane review that requires an ongoing commitment). We have recently (April 2018) improved the platform’s functionality in tracking task matching and completion and look forward to monitoring these metrics going forward.

### User Feedback

All development of the platform has been in response to issues or opportunities identified by users, sometimes through formal user research and sometimes through informal feedback.

Most members report successfully posting tasks and getting rapid, useful responses.

We used TaskExchange late last year and had a quick and positive response. Four articles were translated within a week for one of our Cochrane Reviews on breast reconstruction. TaskExchange is a great platform to speed up what could otherwise be a laborious process of finding people to help on a review.

Melina Willson, Managing Editor, Cochrane Breast Cancer Review Group located at the National Health and Medical Research Council (NHMRC) Clinical Trials Centre, University of Sydney, Australia

Similarly, TaskExchange members, particularly those seeking to build their experience with evidence synthesis, have been very positive about their experiences, often noting that initial small tasks led to more substantive roles.

I had started to work on reviews, and I noticed TaskExchange somewhere on the Cochrane webpage when browsing. I saw that an author team wanted help translating a Polish trial article, so I volunteered to do that, and was acknowledged in the publication which was a bonus for my CV. The authors have offered me more translation work, which is fantastic. I'd like to gain more skills in other aspects of reviewing, and I'm planning to seek opportunities through TaskExchange to meet my learning goals. TaskExchange has made it easy to get involved with more SRs and I’d recommend it to anyone wanting more experience.

Jan Witowski, 4th year medical student from Poland

## Discussion

### Principal Findings

This project demonstrates the potential of a Web-based marketplace platform to facilitate collaboration in health evidence synthesis. TaskExchange has been open access since August 2016, has over 2300 members, and has hosted more than 630 tasks covering a wide range of evidence synthesis activities. The task response rate since August 2016 has been 78%. Our focus is now on further building the TaskExchange community.

### Connections to Other Work

The development of TaskExchange has occurred at a time when commercial Web-based marketplaces, such as Airtasker, 99designs, and Freelancer, are becoming more common in professional environments. However, while there are some exceptions (eg, Open Science Framework and Science Exchange‎), the uptake of Web-based marketplace approaches to connect health research professionals has been relatively slow, and studies of their impact are rare [5]. As a result, while there has been a rapid increase in the use of social networks for health research (eg, for participant recruitment or dissemination of health promotion messages) and research about the effectiveness of these approaches [13,14], we know little about how to use peer-to-peer approaches to support and connect researchers themselves.

There is a clear potential benefit in creating an integrated health evidence ecosystem in which health care decision makers, researchers, knowledge brokers, consumers, and others function as part of one closed-loop system [15]. Evidence suggests that involvement in research by clinicians can improve health care delivery and outcomes [16,17]. Platforms like TaskExchange are a useful first step in this direction, providing an easy entry into the research world for people interested in contributing to health research and also a useful portal for health researchers to reach out to other contributors in the broader health research and health care system. This potential to bring the research and nonresearch worlds closer is reflected in the large number of tasks on TaskExchange seeking contributors from consumers.

### Limitations

As noted, we have limited data on the use and utility of TaskExchange. The focus on building user features and limited administrative or management features has made sense while we have been operating in a prototyping, “can-it-work?” mode. As we focus more on refining, expanding, and sustaining TaskExchange, we will further build these elements of the system to help us better respond to user needs and understand how the platform can be further developed and improved.

### Next Steps

Building on our partnership with the Guidelines International Network, we are also exploring opportunities to expand the use of TaskExchange to other organizations working in health evidence synthesis. Given the similarity of tasks undertaken and the overlap in communities, it seems natural that guideline developers, health technology assessment organizations, and others in similar fields would benefit from TaskExchange. Acknowledging this, TaskExchange has recently been customized and broadened to meet the needs of guideline developers, and further changes are possible in the future.

TaskExchange is also a key element of Cochrane’s work to provide pathways for new contributors to join Cochrane. As part of this work, we are looking to build connections between TaskExchange and Cochrane Crowd. Cochrane Crowd is Cochrane’s citizen science platform, a global community of volunteers who are helping to curate the research needed to support informed decision-making about health care by, for example, identifying randomized controlled trials for inclusion in systematic reviews. Cochrane Crowd currently has almost 7000 members who receive training in simple systematic review-related tasks that underpin Cochrane reviews.

TaskExchange provides a natural next step for Crowd participants who wish to further develop their skills and experience. We have recently released a feature that helps new users identify beginner-level tasks within TaskExchange, and we hope in the future to enable Crowd participants to carry their profile over into TaskExchange so that they can demonstrate their track record and experience.

### Conclusions

The development of TaskExchange demonstrates the potential for Web-based collaboration to improve the efficiency of and facilitate broader involvement in health research. Through a relatively simple platform, TaskExchange provides opportunities for systematic reviewers and guideline developers to get their work completed more quickly and provides an effective pathway for people to join the health evidence synthesis community.

## Appendix

Multimedia Appendix 1 Key webpages on the TaskExchange platform.

## Abbreviations

CEPM: community engagement and partnerships manager
NHMRC: National Health and Medical Research Council
