# Supplementary figures and images for: Facilitating Web-Based Collaboration in Evidence Synthesis (TaskExchange): Development and Analysis
Source: JMIR Res Protoc. 2018 Dec 13;7(12):e188. doi: 10.2196/resprot.9285 (PMC6315246; doi:10.2196/resprot.9285)

## Multimedia Appendix 1. Key webpages on the TaskExchange platform

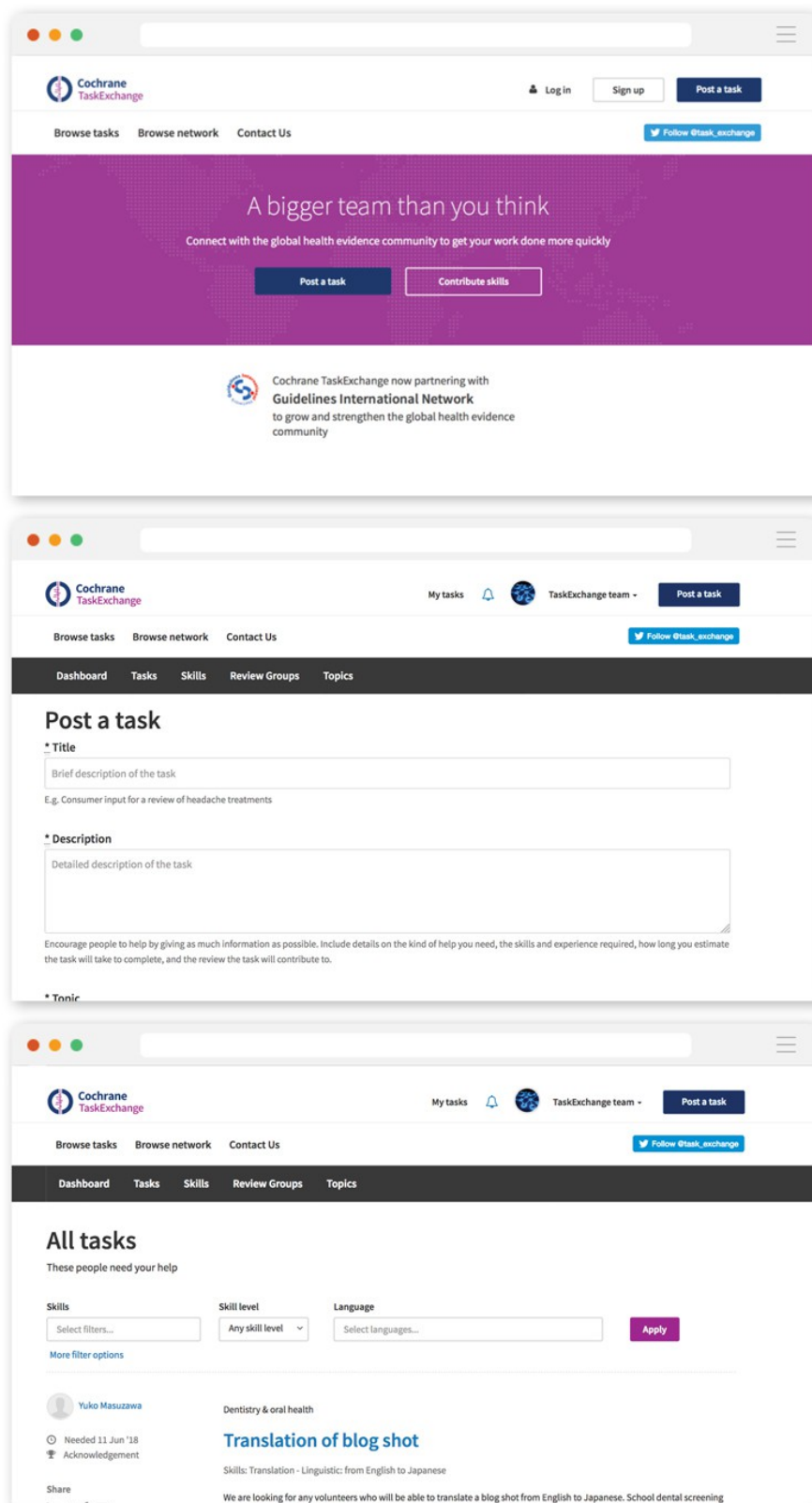

Supplement: Multimedia Appendix 1 [file resprot_v7i12e188_app1.pdf]
